# Supplementary material for: Stepwise assembly of α-hemolysin from intermediates to the mature pore in native erythrocytes
Source: J Cell Biol. 2026 Jan 12;225(3):e202506129. doi: 10.1083/jcb.202506129 (PMC12794805; doi:10.1083/jcb.202506129)
Supplement: Table S1 — shows cryo-EM data collection, image processing, and refinement for pre- and posthemolysis α-HL conformations. [file jcb_202506129_tables1.docx]

**Table S1:** Cryo-EM Data collection, image processing, and refinement for pre- and post-hemolysis α-HL conformations.

| **Data Collection and Processing** | **Post-hemolysis α-HL** **conformations** | **Pre-hemolysis α-HL** **conformations** |  |
| --- | --- | --- | --- |
| Oligomer conformation | Pore | Pre-pore Ia, Ib, II, III, and IV |  |
| Magnification | 54,000 X | 54,000 X |  |
| Voltage | 200 kV | 200 kV |  |
| Electron exposure (e^-^/Å^2^) | 50 | 50 |  |
| Defocus range (µm) | -1.25 to -2.75 | -1.25 to -2.75 |  |
| Pixel size (Å) | 0.92 | 0.92 |  |
| Symmetry Imposed | C7 | C7 |  |
| Number of micrographs | 2102 | 3018 |  |
| Number of particles | 55213 | 44637, 51196, 74616, 50694, and 106804 |  |
| Map Resolution (Å) | 3.1 | 4.6, 4.0, 3.8, 3.8, and 3.4 |  |
| FSC threshold | 0.143 | 0.143 |  |
