# Supplementary material for: Stepwise assembly of α-hemolysin from intermediates to the mature pore in native erythrocytes
Source: J Cell Biol. 2026 Jan 12;225(3):e202506129. doi: 10.1083/jcb.202506129 (PMC12794805; doi:10.1083/jcb.202506129)
Supplement: Table S2 — shows cryo-EM map and model validation for membrane and α-HL complex. [file jcb_202506129_tables2.docx]

**Table S2**: Cryo-EM map and model validation for membrane and α-HL complex.

| **Validation** | **Post-hemolysis pore** | **Pre-hemolysis pre-pore II** | **Pre-hemolysis pre-pore III** | **Pre-hemolysis pre-pore IV** |
| --- | --- | --- | --- | --- |
| MolProbity Score | 2.05 | 2.70 | 2.71 | 1.76 |
| Clash Score | 7.40 | 13.84 | 22.39 | 7.46 |
| Rotamer Outliers (%) | 2.48 | 5.63 | 3.63 | 0.40 |
| Ramachandran Plot |  |  |  |  |
| Outlier (%) | 0.00 | 0.00 | 0.00 | 0.00 |
| Allowed (%) | 4.57 | 7.89 | 6.94 | 5.07 |
| Favored (%) | 95.43 | 92.11 | 93.06 | 94.93 |
