# Supplementary material for: Stepwise assembly of α-hemolysin from intermediates to the mature pore in native erythrocytes
Source: J Cell Biol. 2026 Jan 12;225(3):e202506129. doi: 10.1083/jcb.202506129 (PMC12794805; doi:10.1083/jcb.202506129)
Supplement: Table S3 — shows primer information. [file jcb_202506129_tables3.docx]

**Table S3:** Primer information.

| **Primer Name** | **Primer Sequence** |
| --- | --- |
| **α-HL_179W_FP** | 5’-GGTTAACCAGAACTGGGGTCCGTATGATCG-3’ |
| **α-HL_179W_RP** | 5’-CGATCATACGGACCCCAGTTCTGGTTAACC-3’ |
| **α-HL_200R_FP** | 5’-GCTGTTTATGAAAACCCGTAATGGCAGTATGAAAGC-3’ |
| **α-HL_200R_RP** | 5’-GCTTTCATACTGCCATTACGGGTTTTCATAAACAGC-3’ |
| **α-HL_9RES_FP** | 5’-GGAATTCCATATGGCACCACCGATATTGGCAG-3’ |
| **α-HL_TER_RP** | 5’-CCGCATATGATTGGTCATTTCCTCTTTTTCCC-3’ |
