# Supplementary material for: Stepwise assembly of α-hemolysin from intermediates to the mature pore in native erythrocytes
Source: J Cell Biol. 2026 Jan 12;225(3):e202506129. doi: 10.1083/jcb.202506129 (PMC12794805; doi:10.1083/jcb.202506129)
Supplement: Data S2 — shows values corresponding to the plot related to Fig. 1 D. [file jcb_202506129_datas2.pdf]

Mins	RBC			RBC_α-HL(0.1μM)			RBC_α-HL(0.01μM)			0.001%Triton		
0	1.18849	1.22976	1.21203	1.19704	1.18424	1.21104	1.20236	1.22305	1.20736	0.035875	0.03597	0.035394
0.5	1.18755	1.25754	1.24399	1.17756	1.18309	1.19581	1.2056	1.21234	1.20564	0.036054	0.036141	0.035633
1	1.18307	1.25449	1.2378	1.15318	1.16835	1.17505	1.18883	1.2035	1.19123	0.035962	0.036147	0.035507
1.5	1.18201	1.25285	1.23219	1.1206	1.14103	1.1432	1.17791	1.19502	1.17587	0.035989	0.036196	0.035651
2	1.18734	1.24807	1.22666	1.07388	1.10478	1.11627	1.16384	1.18703	1.16733	0.036001	0.036092	0.035627
2.5	1.1884	1.25187	1.22923	1.02609	1.06557	1.08264	1.1508	1.18332	1.15727	0.036017	0.036072	0.035637
3	1.19682	1.24664	1.22974	0.968711	1.01681	1.03268	1.14301	1.17403	1.15396	0.036269	0.036143	0.03564
3.5	1.20043	1.25308	1.23804	0.913838	0.965699	0.997797	1.14382	1.16661	1.14386	0.036457	0.036204	0.035642
4	1.19997	1.25686	1.24391	0.85716	0.907186	0.955413	1.13292	1.15807	1.13784	0.036327	0.036068	0.035659
4.5	1.20626	1.25484	1.24275	0.811077	0.858095	0.908671	1.12305	1.14356	1.12913	0.036353	0.036228	0.035729
5	1.21087	1.25819	1.24663	0.762514	0.8267	0.853922	1.1163	1.1324	1.12096	0.036437	0.036279	0.035932
5.5	1.20295	1.2521	1.24577	0.705659	0.783518	0.783345	1.10286	1.11255	1.1023	0.036524	0.036371	0.035994
6	1.20443	1.25244	1.24855	0.658867	0.737874	0.727516	1.08572	1.09042	1.08318	0.036478	0.036553	0.035988
6.5	1.20331	1.24999	1.24648	0.613949	0.695245	0.671722	1.06693	1.07466	1.06435	0.03665	0.036683	0.03614
7	1.20012	1.24498	1.24557	0.567971	0.655633	0.625856	1.05346	1.05327	1.04912	0.036474	0.036619	0.036122
7.5	1.19767	1.24377	1.24302	0.521906	0.615684	0.583435	1.03122	1.03426	1.02442	0.036485	0.036679	0.036004
8	1.19459	1.24162	1.24241	0.484645	0.581609	0.530497	1.00858	1.01022	1.00691	0.036456	0.036732	0.036143
8.5	1.19208	1.23858	1.24143	0.438237	0.546651	0.491165	0.994511	0.991948	0.991411	0.036479	0.03673	0.036192
9	1.18735	1.23419	1.23723	0.411407	0.515133	0.453879	0.971198	0.97255	0.972262	0.036408	0.036793	0.036065
9.5	1.18754	1.23159	1.2382	0.367601	0.480825	0.42766	0.953894	0.951831	0.948232	0.036386	0.036691	0.036027
10	1.18806	1.23268	1.23702	0.331277	0.447863	0.400765	0.932883	0.934221	0.935246	0.036348	0.036697	0.035898
10.5	1.18979	1.22967	1.2321	0.308399	0.419716	0.386075	0.912689	0.919494	0.917997	0.036435	0.036635	0.036074
11	1.18652	1.22538	1.23266	0.289552	0.397847	0.345702	0.897595	0.900466	0.905856	0.036436	0.036691	0.03608
11.5	1.18629	1.223	1.23389	0.270784	0.375284	0.322938	0.881502	0.882074	0.887811	0.036285	0.036621	0.036192
12	1.18379	1.22194	1.23184	0.255098	0.35057	0.297982	0.859489	0.865308	0.875275	0.036417	0.036808	0.036156
12.5	1.18418	1.21919	1.22957	0.244824	0.330555	0.286383	0.843767	0.846196	0.850672	0.036348	0.036645	0.036001
13	1.18266	1.21546	1.23049	0.244514	0.30565	0.263554	0.82144	0.826012	0.839535	0.036627	0.036653	0.036092
13.5	1.18038	1.21352	1.22915	0.227438	0.286998	0.23911	0.808385	0.814529	0.822087	0.036289	0.036823	0.036149
14	1.17669	1.20938	1.2279	0.212523	0.271147	0.22458	0.793418	0.801286	0.805636	0.036393	0.03674	0.036287
14.5	1.17416	1.21144	1.22786	0.208875	0.253966	0.208668	0.778314	0.777445	0.791707	0.036436	0.036668	0.03613
15	1.17382	1.2082	1.22493	0.19777	0.234074	0.200324	0.765821	0.77271	0.77563	0.036386	0.03664	0.036125
15.5	1.17534	1.20587	1.2242	0.192117	0.218894	0.199523	0.747399	0.749259	0.753192	0.036435	0.036762	0.036186
16	1.17675	1.20206	1.22325	0.187991	0.203941	0.19528	0.734027	0.724933	0.744774	0.036297	0.036733	0.036131
16.5	1.1718	1.20002	1.22155	0.181248	0.195966	0.189337	0.716934	0.70301	0.730256	0.0364	0.036809	0.036149
17	1.17066	1.19881	1.21884	0.171522	0.190336	0.180075	0.700364	0.689657	0.720886	0.036419	0.036738	0.036178
17.5	1.17329	1.19671	1.21654	0.16754	0.178045	0.171576	0.689318	0.68574	0.700934	0.036508	0.036586	0.036228
18	1.17	1.19557	1.21417	0.163565	0.174368	0.169914	0.671382	0.66569	0.693086	0.036442	0.036701	0.036098
18.5	1.17094	1.19104	1.2108	0.159085	0.170467	0.1634	0.656544	0.646707	0.675624	0.036456	0.036775	0.036134
19	1.17153	1.18891	1.21083	0.151273	0.164219	0.154818	0.649504	0.632296	0.662485	0.036502	0.036753	0.036248
19.5	1.17077	1.18548	1.20866	0.147032	0.158849	0.150608	0.636076	0.618531	0.646949	0.036378	0.036773	0.036179
20	1.17111	1.18459	1.20866	0.144615	0.154849	0.14834	0.624622	0.60283	0.63673	0.03646	0.036644	0.03633
20.5	1.17256	1.18114	1.20625	0.141939	0.15062	0.145472	0.613247	0.590832	0.6212	0.03636	0.036962	0.036164
21	1.17189	1.18317	1.20673	0.141313	0.148196	0.144223	0.601518	0.577191	0.60898	0.036447	0.036829	0.036267
21.5	1.17036	1.17994	1.20369	0.137855	0.146434	0.14103	0.581555	0.564391	0.597072	0.036479	0.036757	0.036164
22	1.1709	1.1798	1.20351	0.136742	0.143444	0.139647	0.57812	0.554183	0.591787	0.036443	0.0367	0.036298
22.5	1.17162	1.17483	1.20224	0.132951	0.14254	0.136397	0.565885	0.539262	0.576632	0.036491	0.036743	0.036383
23	1.16926	1.17279	1.19938	0.131696	0.140375	0.135019	0.553887	0.52413	0.567864	0.036494	0.03674	0.036212
23.5	1.16797	1.17299	1.19723	0.129579	0.13897	0.132947	0.543648	0.513389	0.559013	0.03623	0.03665	0.036382
24	1.16893	1.17209	1.19599	0.126896	0.138413	0.131167	0.533174	0.499119	0.546584	0.036461	0.036735	0.03636
24.5	1.16831	1.16683	1.19627	0.123669	0.137346	0.131045	0.523655	0.487311	0.534616	0.036576	0.036765	0.036389
