# Supplementary material for: Stepwise assembly of α-hemolysin from intermediates to the mature pore in native erythrocytes
Source: J Cell Biol. 2026 Jan 12;225(3):e202506129. doi: 10.1083/jcb.202506129 (PMC12794805; doi:10.1083/jcb.202506129)
Supplement: Data S4 — shows values corresponding to the bar graph related to Fig. 2 G. [file jcb_202506129_datas4.pdf]

α-HL (W179A)			α-HL (W179A-R200A)			α-HL (A1-T9cut)		
24.3	22.6	25.3	5.4	7.5	6.7	19.6	16.6	15.5
