# Supplementary material for: Stepwise assembly of α-hemolysin from intermediates to the mature pore in native erythrocytes
Source: J Cell Biol. 2026 Jan 12;225(3):e202506129. doi: 10.1083/jcb.202506129 (PMC12794805; doi:10.1083/jcb.202506129)
Supplement: Data S5 — shows values corresponding to the plot related to Fig. 4 A. [file jcb_202506129_datas5.pdf]

Mins	RBC_α-HL_37°C(0.1μM)			RBC_α-HL_37°C(0.01μM)			RBC_α-HL_4°C(0.1μM)			RBC_α-HL_4°C(0.01μM)		
0	1.19704	1.18424	1.21104	1.20236	1.22305	1.20736	1.2	1.2	1.2	1.2	1.2	1.2
0.5	1.17756	1.18309	1.19581	1.2056	1.21234	1.20564	1.198819	1.201202	1.202819	1.204526	1.204664	1.20423
1	1.15318	1.16835	1.17505	1.18883	1.2035	1.19123	1.201561	1.203961	1.205844	1.208862	1.209886	1.210735
1.5	1.1206	1.14103	1.1432	1.17791	1.19502	1.17587	1.205263	1.207177	1.207447	1.214639	1.216092	1.215245
2	1.07388	1.10478	1.11627	1.16384	1.18703	1.16733	1.205515	1.208285	1.209365	1.219316	1.221568	1.220662
2.5	1.02609	1.06557	1.08264	1.1508	1.18332	1.15727	1.206856	1.21089	1.211704	1.223827	1.225838	1.224392
3	0.968711	1.01681	1.03268	1.14301	1.17403	1.15396	1.206482	1.210458	1.211646	1.226254	1.23079	1.226962
3.5	0.913838	0.965699	0.997797	1.14382	1.16661	1.14386	1.208578	1.213168	1.213457	1.230088	1.233683	1.229809
4	0.85716	0.907186	0.955413	1.13292	1.15807	1.13784	1.207808	1.211035	1.211142	1.233223	1.233995	1.229736
4.5	0.811077	0.858095	0.908671	1.12305	1.14356	1.12913	1.205104	1.206896	1.204613	1.23514	1.234208	1.22859
5	0.762514	0.8267	0.853922	1.1163	1.1324	1.12096	1.198248	1.198123	1.192064	1.237105	1.232486	1.224114
5.5	0.705659	0.783518	0.783345	1.10286	1.11255	1.1023	1.188878	1.185437	1.175286	1.237598	1.231126	1.219507
6	0.658867	0.737874	0.727516	1.08572	1.09042	1.08318	1.172981	1.167713	1.152464	1.236461	1.227535	1.209745
6.5	0.613949	0.695245	0.671722	1.06693	1.07466	1.06435	1.153838	1.148707	1.129156	1.23483	1.21992	1.195074
7	0.567971	0.655633	0.625856	1.05346	1.05327	1.04912	1.132532	1.125274	1.101175	1.230502	1.208706	1.177735
7.5	0.521906	0.615684	0.583435	1.03122	1.03426	1.02442	1.10757	1.097053	1.06351	1.22319	1.194893	1.156746
8	0.484645	0.581609	0.530497	1.00858	1.01022	1.00691	1.075112	1.057998	1.013336	1.213214	1.178015	1.132068
8.5	0.438237	0.546651	0.491165	0.994511	0.991948	0.991411	1.036765	1.012567	0.957271	1.20436	1.159693	1.104492
9	0.411407	0.515133	0.453879	0.971198	0.97255	0.972262	0.987641	0.957392	0.899387	1.189021	1.13706	1.066828
9.5	0.367601	0.480825	0.42766	0.953894	0.951831	0.948232	0.935926	0.90472	0.845072	1.171909	1.114132	1.029024
10	0.331277	0.447863	0.400765	0.932883	0.934221	0.935246	0.885764	0.850796	0.790686	1.155607	1.085424	0.987276
10.5	0.308399	0.419716	0.386075	0.912689	0.919494	0.917997	0.835969	0.797759	0.737696	1.137875	1.052888	0.944701
11	0.289552	0.397847	0.345702	0.897595	0.900466	0.905856	0.787202	0.745926	0.686812	1.11805	1.017804	0.905858
11.5	0.270784	0.375284	0.322938	0.881502	0.882074	0.887811	0.739999	0.695782	0.636401	1.094716	0.980898	0.86709
12	0.255098	0.35057	0.297982	0.859489	0.865308	0.875275	0.695518	0.647556	0.590617	1.071055	0.945985	0.83309
12.5	0.244824	0.330555	0.286383	0.843767	0.846196	0.850672	0.65269	0.603067	0.547332	1.046099	0.912392	0.798889
13	0.244514	0.30565	0.263554	0.82144	0.826012	0.839535	0.611024	0.559122	0.505933	1.017364	0.879226	0.76579
13.5	0.227438	0.286998	0.23911	0.808385	0.814529	0.822087	0.570757	0.51625	0.466174	0.989153	0.849534	0.733848
14	0.212523	0.271147	0.22458	0.793418	0.801286	0.805636	0.532592	0.477832	0.429282	0.962557	0.820458	0.705035
14.5	0.208875	0.253966	0.208668	0.778314	0.777445	0.791707	0.497436	0.442957	0.397426	0.935516	0.793678	0.677042
15	0.19777	0.234074	0.200324	0.765821	0.77271	0.77563	0.464614	0.408882	0.366293	0.909796	0.767237	0.650977
15.5	0.192117	0.218894	0.199523	0.747399	0.749259	0.753192	0.43516	0.378402	0.338209	0.882523	0.741175	0.622637
16	0.187991	0.203941	0.19528	0.734027	0.724933	0.744774	0.40763	0.353628	0.313458	0.858696	0.714737	0.597106
16.5	0.181248	0.195966	0.189337	0.716934	0.70301	0.730256	0.38072	0.327578	0.29138	0.8316	0.688667	0.571015
17	0.171522	0.190336	0.180075	0.700364	0.689657	0.720886	0.355302	0.306613	0.271932	0.811392	0.667322	0.551933
17.5	0.16754	0.178045	0.171576	0.689318	0.68574	0.700934	0.333702	0.287129	0.255712	0.793422	0.646393	0.531534
18	0.163565	0.174368	0.169914	0.671382	0.66569	0.693086	0.31501	0.270547	0.241622	0.77269	0.625315	0.511219
18.5	0.159085	0.170467	0.1634	0.656544	0.646707	0.675624	0.297728	0.254802	0.228864	0.751418	0.603169	0.488834
19	0.151273	0.164219	0.154818	0.649504	0.632296	0.662485	0.281545	0.24043	0.216649	0.732203	0.583908	0.47102
19.5	0.147032	0.158849	0.150608	0.636076	0.618531	0.646949	0.265246	0.226247	0.206288	0.709762	0.564383	0.45244
20	0.144615	0.154849	0.14834	0.624622	0.60283	0.63673	0.25338	0.215851	0.197838	0.690943	0.545898	0.435355
20.5	0.141939	0.15062	0.145472	0.613247	0.590832	0.6212	0.238493	0.204823	0.188926	0.668564	0.52645	0.418618
21	0.141313	0.148196	0.144223	0.601518	0.577191	0.60898	0.228587	0.196062	0.181819	0.651179	0.510095	0.404215
21.5	0.137855	0.146434	0.14103	0.581555	0.564391	0.597072	0.21769	0.187169	0.176324	0.631781	0.492715	0.388417
22	0.136742	0.143444	0.139647	0.57812	0.554183	0.591787	0.209004	0.180424	0.169609	0.617266	0.480181	0.377279
22.5	0.132951	0.14254	0.136397	0.565885	0.539262	0.576632	0.20065	0.17287	0.163904	0.598565	0.464024	0.362783
23	0.131696	0.140375	0.135019	0.553887	0.52413	0.567864	0.192829	0.166673	0.158905	0.580913	0.448374	0.350742
23.5	0.129579	0.13897	0.132947	0.543648	0.513389	0.559013	0.185903	0.160849	0.154234	0.564767	0.434917	0.340613
24	0.126896	0.138413	0.131167	0.533174	0.499119	0.546584	0.180209	0.155954	0.150305	0.54948	0.421766	0.329032
24.5	0.123669	0.137346	0.131045	0.523655	0.487311	0.534616						
