# Supplementary material for: Stepwise assembly of α-hemolysin from intermediates to the mature pore in native erythrocytes
Source: J Cell Biol. 2026 Jan 12;225(3):e202506129. doi: 10.1083/jcb.202506129 (PMC12794805; doi:10.1083/jcb.202506129)
Supplement: Data S6 — shows values corresponding to the plot related to Fig. 4 C. [file jcb_202506129_datas6.pdf]

Lipid+α-HL Stored at 37°C

	1	2	3	4	5	6	7	8	9	10	11	12
260	0.173236	0.120772	0.167005	0.128066	-1.00385	-1.0024	-0.83894	-0.42629	-0.34168	-0.26414	-0.22569	-0.25724
259.5	0.175716	0.10976	0.160276	0.126395	-1.01526	-1.02331	-0.8475	-0.46217	-0.35066	-0.27026	-0.25252	-0.26132
259	0.201665	0.132037	0.152755	0.120266	-1.02396	-1.03789	-0.848	-0.48447	-0.36029	-0.28742	-0.26781	-0.25253
258.5	0.217309	0.145587	0.130833	0.115497	-1.06337	-1.06398	-0.8849	-0.50408	-0.38688	-0.30838	-0.28162	-0.25983
258	0.237474	0.158142	0.112253	0.122162	-1.083	-1.09519	-0.91584	-0.50421	-0.40435	-0.31841	-0.28374	-0.26763
257.5	0.232245	0.171056	0.118843	0.123025	-1.08523	-1.13274	-0.93075	-0.51981	-0.40996	-0.33884	-0.31559	-0.27352
257	0.233027	0.167677	0.111918	0.14023	-1.12427	-1.17629	-0.95522	-0.53106	-0.43602	-0.35083	-0.33738	-0.2831
256.5	0.252007	0.182917	0.124875	0.144682	-1.168	-1.20992	-0.97152	-0.54096	-0.45698	-0.35122	-0.3598	-0.30716
256	0.25869	0.197592	0.145484	0.153431	-1.19406	-1.23665	-0.99217	-0.56187	-0.47054	-0.36122	-0.39708	-0.32133
255.5	0.264798	0.206533	0.151231	0.147028	-1.2201	-1.27358	-1.02171	-0.60098	-0.49582	-0.38901	-0.41429	-0.34526
255	0.249628	0.218942	0.141972	0.142266	-1.26806	-1.34038	-1.06987	-0.61513	-0.53237	-0.42306	-0.45021	-0.37374
254.5	0.248227	0.224464	0.140738	0.136672	-1.29791	-1.38687	-1.1024	-0.61397	-0.54619	-0.45322	-0.47568	-0.39008
254	0.218829	0.22186	0.120757	0.10369	-1.35869	-1.43545	-1.14845	-0.66446	-0.57984	-0.48813	-0.5262	-0.41972
253.5	0.218376	0.225398	0.118042	0.112537	-1.38422	-1.47804	-1.18646	-0.68427	-0.58415	-0.50388	-0.53642	-0.44124
253	0.224408	0.235389	0.103552	0.116378	-1.44087	-1.5173	-1.22164	-0.68825	-0.61292	-0.52597	-0.55938	-0.46673
252.5	0.224514	0.246712	0.113948	0.134286	-1.48659	-1.55291	-1.25078	-0.68343	-0.62622	-0.53561	-0.57013	-0.49047
252	0.200063	0.222334	0.113135	0.132173	-1.55886	-1.62665	-1.3038	-0.71058	-0.65096	-0.56267	-0.60418	-0.53929
251.5	0.18763	0.20009	0.118314	0.118456	-1.63624	-1.68648	-1.32664	-0.76218	-0.67986	-0.59537	-0.62943	-0.56922
251	0.18784	0.180505	0.115371	0.102861	-1.72134	-1.74784	-1.39405	-0.82158	-0.71954	-0.62381	-0.65722	-0.59971
250.5	0.180016	0.167414	0.109971	0.081332	-1.80052	-1.82152	-1.45634	-0.87708	-0.75098	-0.66476	-0.6883	-0.64642
250	0.190665	0.175418	0.122503	0.079072	-1.86801	-1.87275	-1.48885	-0.90456	-0.75801	-0.69108	-0.70769	-0.6664
249.5	0.182915	0.148085	0.113884	0.066211	-1.94389	-1.95483	-1.56792	-0.9509	-0.77865	-0.72556	-0.71809	-0.68812
249	0.15219	0.095655	0.074104	0.021782	-2.0485	-2.05908	-1.65294	-1.00733	-0.84221	-0.78139	-0.764	-0.75915
248.5	0.138308	0.062865	0.053499	-0.01303	-2.12738	-2.14071	-1.70637	-1.06062	-0.88226	-0.82583	-0.80935	-0.79766
248	0.120564	0.023303	0.018703	-0.05148	-2.20847	-2.23594	-1.79441	-1.12141	-0.92901	-0.865	-0.85166	-0.84346
247.5	0.076209	-0.02972	-0.02203	-0.08434	-2.32074	-2.35319	-1.88753	-1.20354	-0.99496	-0.90628	-0.90905	-0.89155
247	0.01871	-0.08816	-0.07165	-0.14935	-2.45132	-2.49789	-1.98409	-1.29211	-1.08018	-0.96747	-0.99523	-0.98364
246.5	-0.04268	-0.14082	-0.12661	-0.22791	-2.60591	-2.64247	-2.09347	-1.39027	-1.16649	-1.03241	-1.06586	-1.06139
246	-0.1	-0.20239	-0.19605	-0.31966	-2.75799	-2.76638	-2.19146	-1.48717	-1.24679	-1.09541	-1.13757	-1.13276
245.5	-0.14946	-0.25309	-0.26028	-0.39956	-2.90148	-2.90151	-2.29473	-1.55249	-1.32499	-1.15808	-1.20246	-1.20208
245	-0.22267	-0.33	-0.34831	-0.4877	-3.05961	-3.04291	-2.40932	-1.63374	-1.39999	-1.24622	-1.29024	-1.27905
244.5	-0.30652	-0.39018	-0.44284	-0.58825	-3.21281	-3.22011	-2.53203	-1.70094	-1.49459	-1.32966	-1.37375	-1.36367
244	-0.38065	-0.4795	-0.54063	-0.67765	-3.41215	-3.37608	-2.62418	-1.76854	-1.57932	-1.41431	-1.45215	-1.43533
243.5	-0.48437	-0.58375	-0.65548	-0.80256	-3.60667	-3.54893	-2.75861	-1.86092	-1.66993	-1.49425	-1.53054	-1.53027
243	-0.59929	-0.69876	-0.79479	-0.95052	-3.81322	-3.74881	-2.88793	-1.95069	-1.78193	-1.60653	-1.63895	-1.66042
242.5	-0.71	-0.79444	-0.9312	-1.075	-4.02636	-3.93298	-3.02696	-2.05926	-1.8863	-1.72193	-1.74896	-1.78567
242	-0.83432	-0.90805	-1.05372	-1.20548	-4.25461	-4.11612	-3.18367	-2.15836	-1.9653	-1.82845	-1.84951	-1.91028
241.5	-0.96265	-1.03743	-1.18632	-1.33576	-4.52369	-4.30772	-3.32782	-2.27622	-2.07256	-1.9533	-1.9572	-2.05479
241	-1.09087	-1.15678	-1.33633	-1.49021	-4.7933	-4.53556	-3.472	-2.39089	-2.19386	-2.09062	-2.07859	-2.19195
240.5	-1.19463	-1.28769	-1.4753	-1.66225	-5.04139	-4.75731	-3.61684	-2.50218	-2.2968	-2.21283	-2.18845	-2.33036
240	-1.34935	-1.44521	-1.64859	-1.83826	-5.30246	-4.99832	-3.80159	-2.63546	-2.43406	-2.3572	-2.31626	-2.49423
239.5	-1.48304	-1.60701	-1.80954	-2.00333	-5.54726	-5.25675	-3.96552	-2.75768	-2.5768	-2.50791	-2.44388	-2.65836
239	-1.62099	-1.74327	-1.95507	-2.16939	-5.80572	-5.49195	-4.1293	-2.91018	-2.6928	-2.62445	-2.57185	-2.80998
238.5	-1.76023	-1.88955	-2.10341	-2.34272	-6.03783	-5.70586	-4.30993	-3.05503	-2.81301	-2.75454	-2.72451	-2.95517
238	-1.90114	-2.03558	-2.26377	-2.53307	-6.29912	-5.91795	-4.45742	-3.18411	-2.95484	-2.88242	-2.87024	-3.12981
237.5	-2.05357	-2.18202	-2.43169	-2.73544	-6.5409	-6.16214	-4.6298	-3.34231	-3.08411	-3.03193	-3.03281	-3.31474
237	-2.2094	-2.35147	-2.61614	-2.92754	-6.78195	-6.4182	-4.80911	-3.49092	-3.21962	-3.18899	-3.2055	-3.49531
236.5	-2.32319	-2.49799	-2.75359	-3.09261	-7.05586	-6.65938	-4.9672	-3.6219	-3.35131	-3.32253	-3.34074	-3.65235
236	-2.47687	-2.65849	-2.92392	-3.28075	-7.35966	-6.88106	-5.153	-3.75119	-3.50608	-3.44902	-3.50665	-3.80771
235.5	-2.60847	-2.80641	-3.08563	-3.47706	-7.64381	-7.08127	-5.30603	-3.89543	-3.6258	-3.56129	-3.6548	-3.96282
235	-2.76103	-2.95868	-3.26877	-3.70699	-7.88719	-7.33163	-5.46794	-4.04671	-3.76414	-3.6834	-3.79196	-4.11708
234.5	-2.92711	-3.16945	-3.45693	-3.94115	-8.13295	-7.55016	-5.66914	-4.18211	-3.88501	-3.78929	-3.94598	-4.26012
234	-3.11314	-3.34919	-3.65777	-4.15271	-8.37128	-7.71341	-5.83393	-4.30097	-4.01136	-3.90668	-4.09352	-4.4345
233.5	-3.30068	-3.55456	-3.84756	-4.38246	-8.58452	-7.89421	-5.9816	-4.39388	-4.11496	-4.01399	-4.25009	-4.58518
233	-3.51954	-3.78201	-4.07739	-4.64881	-8.86266	-8.12136	-6.13696	-4.54677	-4.23189	-4.11678	-4.43325	-4.75214
232.5	-3.80337	-4.08239	-4.34838	-4.97533	-9.17475	-8.3734	-6.33868	-4.71311	-4.38592	-4.2581	-4.61879	-4.91044
232	-4.12257	-4.3903	-4.68635	-5.32433	-9.52221	-8.64998	-6.52519	-4.87234	-4.54651	-4.43038	-4.78361	-5.12018
231.5	-4.45459	-4.75367	-5.02292	-5.68395	-9.8566	-8.89944	-6.73041	-5.02652	-4.69008	-4.58887	-4.92304	-5.291
231	-4.82002	-5.14037	-5.38701	-6.09991	-10.1787	-9.12458	-6.9716	-5.18787	-4.83734	-4.73127	-5.07585	-5.44972
230.5	-5.24544	-5.59159	-5.81673	-6.54498	-10.5208	-9.40121	-7.19555	-5.35365	-5.02025	-4.89077	-5.24354	-5.63837
230	-5.74419	-6.04046	-6.3299	-7.01896	-10.8131	-9.6435	-7.41244	-5.55364	-5.17912	-5.04322	-5.44697	-5.81428
229.5	-6.28361	-6.56866	-6.88769	-7.54046	-11.1103	-9.93526	-7.62905	-5.76901	-5.34001	-5.18801	-5.63249	-5.99089
229	-6.88095	-7.17432	-7.46445	-8.13622	-11.466	-10.2927	-7.87413	-5.97046	-5.53232	-5.38813	-5.80877	-6.18328
228.5	-7.52184	-7.85103	-8.10596	-8.74828	-11.8708	-10.6473	-8.15666	-6.18104	-5.70534	-5.60124	-5.98717	-6.39162
228	-8.24972	-8.55532	-8.8492	-9.411	-12.2945	-11.0094	-8.41652	-6.4442	-5.89924	-5.79311	-6.1811	-6.56954
227.5	-9.05803	-9.37242	-9.67697	-10.1978	-12.7647	-11.4411	-8.68827	-6.73676	-6.10475	-6.02459	-6.41193	-6.78433
227	-9.96133	-10.2571	-10.5548	-11.0343	-13.2902	-11.851	-8.96234	-7.03711	-6.36193	-6.26608	-6.66138	-7.00169
226.5	-10.9024	-11.1553	-11.5	-11.9007	-13.859	-12.2202	-9.29967	-7.33285	-6.61688	-6.51144	-6.86465	-7.21026
226	-11.9069	-12.1201	-12.4922	-12.8068	-14.4165	-12.6214	-9.6443	-7.60058	-6.85553	-6.75184	-7.0484	-7.40375
225.5	-13.0158	-13.1571	-13.5412	-13.7943	-14.9572	-13.0588	-10.003	-7.88364	-7.11726	-6.99451	-7.24146	-7.62691
225	-14.1673	-14.2816	-14.5993	-14.8505	-15.482	-13.4102	-10.3383	-8.15421	-7.4071	-7.21294	-7.44107	-7.8381
224.5	-15.4406	-15.4476	-15.7381	-15.8702	-16.0547	-13.829	-10.668	-8.41665	-7.70989	-7.46103	-7.64785	-8.05122
224	-16.7105	-16.6995	-16.941	-16.9721	-16.5986	-14.2304	-10.9784	-8.68915	-7.9704	-7.73897	-7.83388	-8.25128
223.5	-18.0512	-18.0434	-18.1653	-18.1419	-17.253	-14.6133	-11.3007	-8.98103	-8.29123	-8.03208	-8.10083	-8.41622
223	-19.4247	-19.3783	-19.4256	-19.4248	-17.8695	-15.0021	-11.6038	-9.30636	-8.58869	-8.33294	-8.35096	-8.63466
222.5	-20.7999	-20.7353	-20.5937	-20.6644	-18.4928	-15.3069	-11.9335	-9.60488	-8.85389	-8.5997	-8.5574	-8.79569
222	-22.2216	-22.0918	-21.8297	-21.9217	-19.0107	-15.5812	-12.1686	-9.86021	-9.14594	-8.74469	-8.81773	-8.89253
221.5	-23.6095	-23.4435	-23.0785	-23.1917	-19.3851	-15.9132	-12.376	-10.0727	-9.41206	-8.94279	-9.08481	-9.01098
221	-24.9217	-24.5845	-24.2789	-24.3234	-19.7762	-16.1124	-12.6378	-10.2804	-9.66782	-9.07483	-9.29639	-9.13355
220.5	-26.2347	-25.7848	-25.5153	-25.3319	-20.0799	-16.2816	-12.8663	-10.5469	-9.92676	-9.23691	-9.46356	-9.27711
220	-27.4669	-27.0892	-26.7182	-26.2762	-20.2137	-16.4494	-13.0133	-10.7806	-10.1476	-9.43121	-9.62726	-9.37641
219.5	-28.5911	-28.4215	-27.85	-27.0892	-20.3591	-16.6341	-13.0601	-10.9385	-10.3485	-9.60102	-9.67608	-9.47596
219	-29.6574	-29.5315	-28.761	-27.864	-20.5383	-16.6858	-13.1206	-10.9964	-10.4686	-9.70024	-9.72118	-9.56981
218.5	-30.6808	-30.3421	-29.6175	-28.5824	-20.4694	-16.6697	-13.1972	-11.1516	-10.5088	-9.78664	-9.7587	-9.62105
218	-31.6521	-31.2277	-30.5302	-29.3348	-20.6121	-16.7074	-13.3046	-11.2463	-10.5797	-9.84378	-9.84315	-9.7088
217.5	-32.5689	-31.865	-31.0712	-29.9879	-20.5935	-16.5461	-13.3262	-11.4588	-10.7255	-10.0125	-9.96098	-9.70856
217	-33.1831	-32.3215	-31.5051	-30.5066	-20.3683	-16.5857	-13.2639	-11.5565	-10.675	-10.1364	-9.98885	-9.68824
216.5	-33.5947	-32.7163	-31.934	-30.8778	-19.906	-16.3169	-13.1722	-11.7248	-10.7078	-10.1402	-9.95923	-9.55151
216	-33.9508	-32.9466	-32.1144	-30.7996	-19.4669	-15.8902	-12.8965	-11.7223	-10.7302	-10.1814	-9.96728	-9.5278
215.5	-34.2155	-32.9901	-32.469	-30.4857	-18.8513	-15.6367	-12.8746	-11.7825	-10.7054	-10.3721	-9.9218	-9.5543
215	-34.4628	-33.0298	-32.5534	-30.3782	-18.3476	-15.5086	-12.8568	-11.7517	-10.682	-10.3983	-9.96837	-9.65322
214.5	-34.6843	-33.2203	-32.574	-30.3247	-17.7658	-15.2839	-12.7425	-11.6681	-10.736	-10.3621	-9.8206	-9.82526
214	-34.4403	-32.9174	-32.4349	-30.324	-17.1958	-14.9977	-12.2631	-11.3155	-10.6021	-10.3335	-9.55746	-9.67443
213.5	-34.0589	-32.617	-32.2712	-29.9985	-16.906	-14.8307	-12.3578	-11.0352	-10.2458	-10.067	-9.42598	-9.76617
213	-33.7499	-31.9667	-31.8811	-29.334	-16.6948	-14.0754	-12.1799	-10.8533	-10.0657	-9.65637	-9.30855	-9.83828
212.5	-33.4149	-31.2222	-31.8625	-29.0959	-15.8711	-13.5629	-11.511	-10.6462	-9.89559	-9.49369	-9.32505	-9.73196
212	-33.0513	-30.2927	-30.8109	-28.8533	-15.3807	-13.0254	-11.222	-10.2706	-9.84073	-9.49904	-9.19873	-9.63751
211.5	-32.6013	-28.8946	-30.048	-28.2802	-14.7031	-12.1633	-10.9449	-10.0852	-9.62591	-9.41302	-9.04745	-9.41595
211	-31.1931	-27.4637	-28.756	-27.2252	-13.1773	-11.6546	-10.9316	-10.0429	-9.18783	-9.20667	-8.93769	-9.56748
210.5	-30.1198	-26.2304	-28.0435	-27.0662	-11.9224	-10.2174	-10.6241	-9.41219	-9.06681	-9.1235	-8.84098	-9.54199
210	-28.6647	-24.8744	-26.6965	-25.6084	-10.423	-9.92711	-10.3748	-9.10911	-8.53473	-8.88122	-8.67549	-9.55199
209.5	-27.096	-23.3975	-24.9885	-24.8353	-10.7046	-9.48832	-10.3007	-8.49078	-8.1636	-8.88408	-8.30715	-10.0989
209	-26.5834	-22.1318	-23.2881	-23.2416	-10.9213	-8.71899	-9.76096	-8.55739	-7.93239	-9.25204	-8.13914	-10.7985
208.5	-25.4807	-21.7349	-21.0958	-21.5083	-9.25804	-7.40117	-9.5375	-7.19115	-8.27505	-8.89249	-8.02941	-11.266
208	-22.3728	-19.8883	-19.0866	-19.9628	-8.15713	-6.53852	-8.29864	-6.76646	-7.86507	-8.22003	-7.79246	-11.82
207.5	-19.8059	-18.5334	-17.9219	-18.5352	-7.00134	-5.36109	-7.71822	-6.18791	-7.52989	-8.16845	-7.58876	-11.7395
207	-18.2557	-16.362	-16.3301	-16.7629	-6.99887	-4.64772	-6.539	-5.94543	-7.47229	-8.28122	-7.43351	-11.1309
206.5	-17.345	-14.5374	-15.7404	-14.4418	-6.61161	-4.95923	-5.58974	-5.12953	-7.22574	-9.3576	-6.91218	-10.154
206	-15.581	-12.7243	-14.0766	-11.8074	-5.29605	-4.83865	-5.55332	-4.56985	-6.12826	-10.3111	-6.76548	-9.57499
205.5	-12.9424	-10.5917	-12.9067	-10.4547	-4.95936	-6.54804	-5.82499	-5.65172	-6.46825	-9.00789	-4.68881	-8.78488
205	-10.8409	-9.93661	-10.8035	-9.31898	-3.76978	-6.19335	-6.88951	-5.32701	-6.63437	-8.30519	-5.76514	-6.7062
204.5	-8.46302	-9.24549	-9.36488	-7.62899	-3.88478	-6.23395	-7.75383	-4.37544	-5.30485	-8.1024	-5.02551	-6.97159
204	-5.27208	-7.68612	-8.90739	-6.74088	-2.99543	-6.0542	-7.42934	-4.84844	-6.29475	-7.4415	-4.30813	-7.28278
203.5	-4.35914	-6.20427	-7.70591	-6.33633	-2.82497	-6.83167	-4.66226	-5.24075	-5.84656	-7.95849	-3.834	-7.21517
203	-3.35784	-7.01686	-6.30436	-6.0809	-3.52206	-6.84482	-4.45704	-5.59665	-5.80861	-9.27203	-3.32027	-6.94544
202.5	-2.00928	-6.1488	-5.97283	-7.02939	-2.22403	-5.31363	-4.76291	-6.35897	-4.49939	-7.22094	-3.17641	-4.94006
202	-1.70438	-2.37794	-6.04998	-7.41387	-2.70528	-4.08517	-5.62642	-7.29556	-3.37738	-7.93818	-2.80679	-5.49327
201.5	-2.81846	-1.01767	-6.67359	-6.8846	-2.0892	-3.97009	-6.88018	-7.02676	-3.16417	-9.32538	-4.55113	-2.81856
201	-3.98853	-0.08483	-7.19282	-8.28972	-1.38819	-3.69947	-6.29967	-7.30680	-2.70767	-8.19942	-4.56875	-0.46869
200.5	-4.97929	1.80606	-5.85322	-8.52637	-2.0022	-3.03334	-6.30135	-8.99215	-3.03151	-7.19718	-6.12177	-0.76137
200	-2.36381	3.16257	-5.49069	-8.49402	-0.79629	-3.47779	-5.8789	-10.3826	-3.15031	-7.2512	-6.10394	-0.49953
199.5	-0.39839	3.3785	-4.44955	-10.5778	-1.77449	-2.50706	-6.97156	-11.8826	-4.06277	-7.08704	-7.48063	1.78983
199	-0.55762	3.79402	-4.75273	-12.6363	-1.07842	-0.60906	-7.73252	-12.2002	-5.59276	-7.01401	-9.61668	3.29928
198.5	-0.31309	2.99434	-6.37063	-12.0374	-0.68112	0.656522	-7.80873	-9.33599	-3.83613	-7.41878	-9.51914	1.83137
198	-0.64821	3.86845	-7.37294	-11.7038	-1.6993	-0.40579	-6.97675	-9.8516	-4.75057	-7.76696	-8.96409	0.174699
197.5	-2.95334	4.99357	-6.68883	-12.0988	-2.7895	0.163771	-7.02017	-9.7292	-4.26155	-8.16044	-9.62702	1.09548
197	-4.17466	4.62843	-7.57093	-12.4529	-4.85553	1.28647	-9.10656	-8.56919	-4.65509	-8.89376	-9.47327	1.95902
196.5	-5.14935	5.96216	-8.37097	-13.3508	-3.82854	1.82086	-11.4771	-7.27022	-3.99596	-9.10976	-9.71425	2.73917
196	-2.99635	7.06875	-10.1133	-12.0969	-3.64337	0.755299	-11.1058	-7.01089	-5.94028	-10.802	-12.6412	0.452063
195.5	-1.58941	6.41755	-9.04577	-12.2057	-1.92683	-1.58324	-7.73774	-7.57926	-8.21327	-10.8972	-12.7457	3.15517
195	-0.97539	5.16828	-7.73848	-11.1912	-1.8821	-2.05587	-6.89741	-6.52141	-9.40965	-10.5343	-11.1959	3.87756

Lipid+α-HL Stored at 4°C

	1	2	3	4	5	6	7	8	9	10	11	12
260	0.059861	0.104456	0.103676	0.037412	-0.6541	-0.81098	-0.85873	-0.80535	-0.59649	-0.51822	-0.32562	-0.27203
259.5	0.070175	0.101313	0.097109	0.026004	-0.67137	-0.84123	-0.87714	-0.81488	-0.60993	-0.53478	-0.34564	-0.28125
259	0.073278	0.122736	0.107436	0.035337	-0.67642	-0.8751	-0.90105	-0.82203	-0.63553	-0.54945	-0.34548	-0.28475
258.5	0.067196	0.114751	0.101817	0.041765	-0.69278	-0.88911	-0.92447	-0.84088	-0.64417	-0.5736	-0.34629	-0.28465
258	0.075465	0.113211	0.120259	0.040789	-0.7238	-0.91144	-0.93516	-0.87667	-0.6396	-0.56844	-0.34374	-0.28671
257.5	0.070726	0.11092	0.113449	0.028281	-0.75347	-0.93676	-0.9639	-0.9014	-0.64933	-0.58134	-0.33651	-0.28156
257	0.073809	0.124166	0.116805	0.028751	-0.78283	-0.95163	-0.9899	-0.93188	-0.6416	-0.59257	-0.34378	-0.31242
256.5	0.084439	0.143781	0.116799	0.05485	-0.81099	-0.96747	-1.0108	-0.95483	-0.65267	-0.59313	-0.35406	-0.31399
256	0.09502	0.149075	0.1221	0.075547	-0.84327	-0.99601	-1.02896	-0.98347	-0.66971	-0.60048	-0.35765	-0.33343
255.5	0.08523	0.154941	0.120293	0.078497	-0.86627	-1.03821	-1.06094	-1.00432	-0.69368	-0.61667	-0.35653	-0.32142
255	0.071476	0.131707	0.094003	0.064843	-0.90189	-1.0804	-1.10474	-1.04008	-0.72626	-0.63673	-0.36543	-0.34598
254.5	0.088834	0.13452	0.10286	0.063606	-0.91009	-1.10961	-1.14258	-1.05931	-0.73651	-0.6349	-0.36172	-0.36177
254	0.092704	0.141576	0.086339	0.0246	-0.97775	-1.16942	-1.21493	-1.12251	-0.76619	-0.67141	-0.38217	-0.39725
253.5	0.112016	0.1396	0.087723	0.025603	-1.01452	-1.21081	-1.25579	-1.14774	-0.78917	-0.69128	-0.40095	-0.42287
253	0.106877	0.131704	0.094717	0.03011	-1.08562	-1.26781	-1.31813	-1.17919	-0.82136	-0.71686	-0.4082	-0.45006
252.5	0.111277	0.130021	0.088949	0.047569	-1.13039	-1.27841	-1.36243	-1.19416	-0.83842	-0.72682	-0.41489	-0.46415
252	0.113805	0.12286	0.091422	0.032388	-1.1867	-1.33581	-1.41405	-1.24428	-0.8755	-0.75082	-0.47307	-0.50414
251.5	0.104566	0.131403	0.081545	0.02509	-1.22485	-1.38797	-1.47174	-1.2841	-0.91689	-0.79445	-0.51292	-0.54707
251	0.11202	0.123242	0.064817	0.022642	-1.26241	-1.44739	-1.52605	-1.33477	-0.97417	-0.82357	-0.55771	-0.59764
250.5	0.109848	0.111254	0.05701	0.01724	-1.32228	-1.51461	-1.5924	-1.38424	-1.03947	-0.84159	-0.60828	-0.63064
250	0.112329	0.120507	0.059465	0.011641	-1.38433	-1.55479	-1.62321	-1.43496	-1.07138	-0.86309	-0.63921	-0.63001
249.5	0.099282	0.1013	0.037684	-0.00791	-1.43818	-1.62472	-1.69816	-1.46321	-1.1034	-0.90625	-0.67115	-0.65125
249	0.069872	0.056676	-0.00923	-0.0571	-1.5368	-1.70364	-1.8044	-1.54144	-1.16574	-0.98613	-0.73915	-0.73982
248.5	0.07904	0.04605	-0.03667	-0.09696	-1.60213	-1.78299	-1.87038	-1.62809	-1.21554	-1.03246	-0.7963	-0.78895
248	0.057509	0.025501	-0.05937	-0.12129	-1.69703	-1.88401	-1.95521	-1.71216	-1.29625	-1.08583	-0.8671	-0.84109
247.5	0.02193	-0.04577	-0.08995	-0.14044	-1.79337	-1.98314	-2.04904	-1.79885	-1.38115	-1.13893	-0.94612	-0.89325
247	-0.04504	-0.10028	-0.15017	-0.18875	-1.90656	-2.08941	-2.13962	-1.89443	-1.47627	-1.21736	-1.03016	-0.97089
246.5	-0.10152	-0.13878	-0.21704	-0.24043	-2.00251	-2.20377	-2.24393	-2.00964	-1.55511	-1.28241	-1.11205	-1.06647
246	-0.14988	-0.19067	-0.27825	-0.29578	-2.12031	-2.33262	-2.34406	-2.14098	-1.63897	-1.35713	-1.19031	-1.14312
245.5	-0.20412	-0.22606	-0.33282	-0.35301	-2.23585	-2.45946	-2.46934	-2.24793	-1.72195	-1.43043	-1.26404	-1.21688
245	-0.26654	-0.32012	-0.4032	-0.43483	-2.38878	-2.60131	-2.61101	-2.37015	-1.81933	-1.51744	-1.33253	-1.30724
244.5	-0.33182	-0.39289	-0.47249	-0.5098	-2.54812	-2.73421	-2.75124	-2.49498	-1.90508	-1.60928	-1.41631	-1.3895
244	-0.40754	-0.45139	-0.542	-0.58634	-2.68783	-2.88416	-2.89195	-2.62105	-1.98999	-1.71635	-1.50991	-1.48068
243.5	-0.47935	-0.54753	-0.63485	-0.68113	-2.85468	-3.05571	-3.04685	-2.74814	-2.09352	-1.83355	-1.61554	-1.60439
243	-0.56537	-0.64446	-0.71275	-0.79551	-3.0334	-3.22178	-3.21852	-2.91214	-2.23206	-1.95615	-1.75783	-1.73091
242.5	-0.65368	-0.7298	-0.78561	-0.89757	-3.18273	-3.39091	-3.35306	-3.05562	-2.35131	-2.05811	-1.88191	-1.84565
242	-0.77957	-0.8086	-0.88686	-1.00776	-3.34737	-3.57391	-3.51363	-3.20209	-2.47289	-2.18414	-1.99721	-1.95671
241.5	-0.9062	-0.91697	-1.01122	-1.12616	-3.55825	-3.73884	-3.71165	-3.36979	-2.61512	-2.32786	-2.12901	-2.09448
241	-1.03389	-1.02008	-1.14194	-1.26468	-3.77101	-3.92333	-3.8907	-3.5487	-2.727	-2.46693	-2.2608	-2.23045
240.5	-1.14447	-1.12764	-1.24605	-1.40303	-3.96853	-4.11243	-4.06618	-3.71396	-2.8492	-2.59162	-2.38728	-2.37199
240	-1.28711	-1.27253	-1.37386	-1.57729	-4.22359	-4.3542	-4.26337	-3.91468	-2.99473	-2.73387	-2.54037	-2.52687
239.5	-1.40734	-1.42531	-1.50612	-1.75341	-4.46907	-4.53831	-4.46705	-4.07802	-3.13577	-2.89537	-2.69054	-2.65836
239	-1.51817	-1.56494	-1.62169	-1.90155	-4.69097	-4.73411	-4.66711	-4.24601	-3.28513	-3.04486	-2.82993	-2.80931
238.5	-1.62921	-1.68699	-1.77644	-2.06083	-4.92774	-4.94808	-4.85595	-4.4176	-3.41701	-3.18459	-2.97463	-2.98277
238	-1.75417	-1.82636	-1.93391	-2.20829	-5.14964	-5.18065	-5.03046	-4.59622	-3.57137	-3.31936	-3.12334	-3.15049
237.5	-1.90063	-1.97896	-2.09512	-2.38947	-5.41825	-5.4089	-5.25169	-4.77187	-3.75148	-3.47759	-3.27863	-3.32489
237	-2.04846	-2.13277	-2.2642	-2.57896	-5.67634	-5.65789	-5.47406	-4.98697	-3.9381	-3.6339	-3.43191	-3.48883
236.5	-2.20744	-2.26064	-2.42581	-2.75142	-5.93116	-5.89932	-5.67142	-5.16892	-4.08565	-3.77799	-3.5754	-3.66421
236	-2.36014	-2.4279	-2.60343	-2.93587	-6.21883	-6.16194	-5.89874	-5.32488	-4.25667	-3.93374	-3.72287	-3.84832
235.5	-2.48083	-2.58317	-2.75329	-3.09763	-6.48736	-6.40075	-6.06639	-5.46321	-4.39883	-4.09234	-3.8554	-4.01405
235	-2.6159	-2.73768	-2.91049	-3.25626	-6.73614	-6.64333	-6.30907	-5.63588	-4.53103	-4.22628	-4.0044	-4.17482
234.5	-2.76321	-2.90371	-3.09767	-3.44881	-6.99763	-6.9191	-6.52992	-5.79003	-4.70589	-4.38058	-4.14851	-4.34241
234	-2.91941	-3.07766	-3.26385	-3.6509	-7.27146	-7.1781	-6.73086	-5.93914	-4.85699	-4.52297	-4.30858	-4.51539
233.5	-3.10537	-3.26747	-3.459	-3.83171	-7.5261	-7.40146	-6.91366	-6.08728	-4.99152	-4.65298	-4.44563	-4.68784
233	-3.28965	-3.46988	-3.65303	-4.04144	-7.84464	-7.64561	-7.13419	-6.27094	-5.15019	-4.79069	-4.59718	-4.88802
232.5	-3.54116	-3.70085	-3.89613	-4.30787	-8.18979	-7.95801	-7.37177	-6.51122	-5.3265	-4.95342	-4.78616	-5.10931
232	-3.8343	-3.99241	-4.18836	-4.67015	-8.54806	-8.24211	-7.61058	-6.75386	-5.51367	-5.14258	-5.00005	-5.28872
231.5	-4.14412	-4.29097	-4.55408	-5.09981	-8.87516	-8.49603	-7.87059	-6.92714	-5.69635	-5.33176	-5.18777	-5.47131
231	-4.4616	-4.59521	-4.77591	-5.17618	-9.17344	-8.77956	-8.1119	-7.14997	-5.86703	-5.47403	-5.36938	-5.65219
230.5	-4.8225	-4.94759	-5.13494	-5.50328	-9.50658	-9.091	-8.36447	-7.3753	-6.01311	-5.65311	-5.5752	-5.84297
230	-5.21473	-5.36477	-5.55507	-5.86399	-9.85357	-9.41378	-8.66825	-7.61102	-6.21106	-5.84632	-5.76412	-6.05152
229.5	-5.65302	-5.81245	-5.98873	-6.30748	-10.1706	-9.74712	-8.95876	-7.86139	-6.40659	-6.03709	-5.95807	-6.24159
229	-6.14271	-6.27937	-6.47387	-6.79491	-10.564	-10.0976	-9.28509	-8.09419	-6.62166	-6.22212	-6.14397	-6.43901
228.5	-6.66371	-6.79109	-7.01351	-7.33344	-10.9996	-10.4117	-9.61006	-8.36019	-6.84017	-6.45313	-6.35195	-6.63991
228	-7.23316	-7.37246	-7.56396	-7.90684	-11.409	-10.7559	-9.88409	-8.61159	-7.04136	-6.67786	-6.551	-6.84664
227.5	-7.87104	-7.99597	-8.19382	-8.5181	-11.8153	-11.0605	-10.2463	-8.8922	-7.28142	-6.91042	-6.75109	-7.05667
227	-8.54763	-8.68563	-8.89417	-9.19938	-12.2064	-11.4668	-10.5938	-9.16174	-7.54105	-7.18887	-6.99492	-7.25122
226.5	-9.33824	-9.3991	-9.62027	-9.95259	-12.5798	-11.8437	-10.9215	-9.45997	-7.80635	-7.46093	-7.25947	-7.46502
226	-10.1746	-10.1863	-10.4206	-10.7535	-13.0307	-12.2199	-11.2255	-9.75122	-8.04835	-7.75185	-7.48661	-7.70616
225.5	-11.045	-10.989	-11.2366	-11.5711	-13.5034	-12.5758	-11.5385	-10.0184	-8.32686	-8.04349	-7.67815	-7.94364
225	-11.9289	-11.8353	-12.1196	-12.4054	-13.9174	-12.9657	-11.8776	-10.3198	-8.61057	-8.31045	-7.87659	-8.1565
224.5	-12.861	-12.7325	-12.9871	-13.2345	-14.4738	-13.3386	-12.1694	-10.5699	-8.90629	-8.56635	-8.10972	-8.36085
224	-13.8196	-13.717	-13.8864	-14.1325	-15.0116	-13.6706	-12.4521	-10.7759	-9.15522	-8.84848	-8.2788	-8.58924
223.5	-14.8066	-14.6866	-14.8122	-15.1128	-15.4865	-14.0195	-12.7026	-11.026	-9.39813	-9.08464	-8.48019	-8.76653
223	-15.8042	-15.7081	-15.8257	-16.0694	-15.9672	-14.3584	-12.9752	-11.2815	-9.66015	-9.3342	-8.68954	-8.92486
222.5	-16.7812	-16.7354	-16.8062	-16.971	-16.3649	-14.6365	-13.2258	-11.5176	-9.93248	-9.60435	-8.88715	-9.06532
222	-17.8361	-17.8407	-17.7757	-17.9088	-16.7391	-14.9968	-13.4046	-11.7004	-10.141	-9.87017	-9.06541	-9.22549
221.5	-18.8996	-18.877	-18.761	-18.8494	-17.1685	-15.1811	-13.7045	-11.8766	-10.2891	-10.0177	-9.22577	-9.37845
221	-19.9342	-19.8627	-19.6888	-19.7416	-17.4739	-15.3907	-13.8886	-12.0965	-10.4448	-10.1921	-9.39494	-9.47596
220.5	-20.9183	-20.7782	-20.612	-20.5932	-17.6832	-15.6119	-13.9489	-12.1076	-10.5548	-10.3084	-9.50854	-9.59319
220	-21.8006	-21.7058	-21.5071	-21.3887	-17.8908	-15.9205	-14.1441	-12.6742	-10.6625	-10.4087	-9.57217	-9.76306
219.5	-22.6657	-22.5811	-22.2928	-22.1303	-18.0359	-16.1084	-14.285	-12.8319	-10.8031	-10.48	-9.59026	-9.81616
219	-23.445	-23.4061	-23.107	-22.8904	-18.221	-16.1854	-14.3574	-12.9316	-10.8616	-10.5356	-9.63908	-9.85772
218.5	-24.1545	-24.2205	-23.7464	-23.5569	-18.3799	-16.1749	-14.4573	-13.0198	-10.8295	-10.5212	-9.6892	-9.99226
218	-24.7929	-24.9182	-24.3573	-24.075	-18.2343	-16.2032	-14.5746	-13.0884	-10.8306	-10.5071	-9.70227	-10.0397
217.5	-25.3359	-25.5116	-24.9127	-24.573	-18.2531	-16.261	-14.5666	-13.1762	-10.7614	-10.4532	-9.70935	-10.0082
217	-25.795	-25.9373	-25.2997	-25.0293	-18.0984	-16.1744	-14.4439	-13.1099	-10.6975	-10.4705	-9.68848	-10.0113
216.5	-26.3047	-26.2315	-25.5441	-25.2064	-17.9403	-15.9541	-14.1863	-13.0144	-10.5995	-10.4572	-9.70933	-10.0828
216	-26.721	-26.6494	-25.6638	-25.3659	-17.7587	-15.6952	-14.0557	-12.8477	-10.528	-10.3344	-9.66822	-10.0549
215.5	-26.8808	-26.7381	-25.7692	-25.5754	-17.4053	-15.5164	-13.8973	-12.8104	-10.4803	-10.258	-9.63358	-10.0648
215	-26.9228	-26.7581	-25.7252	-25.5602	-16.7877	-15.2212	-13.6579	-12.5579	-10.4108	-10.1558	-9.45752	-10.1176
214.5	-26.9673	-26.9075	-25.7535	-25.5127	-16.3952	-14.8124	-13.1104	-12.5977	-10.3585	-10.085	-9.42086	-10.0751
214	-26.6882	-26.8069	-25.6666	-25.3819	-16.3747	-14.476	-12.7485	-12.2247	-10.3468	-10.0185	-9.38252	-10.1625
213.5	-26.5656	-26.8838	-25.3527	-24.888	-16.1992	-14.2065	-12.4062	-11.7808	-10.3366	-9.97568	-9.38978	-10.1195
213	-26.1734	-26.786	-25.1424	-24.6036	-16.0066	-13.6722	-12.2028	-11.3637	-10.0625	-9.85542	-9.37692	-9.94951
212.5	-25.5609	-26.1687	-24.9681	-24.1765	-15.6539	-13.2926	-12.0372	-10.9213	-9.83407	-9.66366	-9.40803	-10.0167
212	-24.9906	-25.7108	-24.5101	-23.7659	-15.1292	-12.7585	-11.844	-10.4772	-9.67779	-9.52822	-9.58839	-9.93769
211.5	-24.2399	-25.0491	-24.2491	-23.1601	-14.5549	-12.2458	-11.4562	-10.0092	-9.44837	-9.29483	-9.67259	-9.91462
211	-23.231	-24.7296	-23.6523	-22.4302	-13.994	-11.5703	-10.5664	-9.54097	-9.04489	-9.06552	-9.64025	-9.73615
210.5	-22.2634	-24.6848	-23.0089	-21.7711	-13.8185	-10.9487	-10.3598	-9.47046	-8.89425	-8.72683	-9.68764	-9.40425
210	-21.7099	-24.29	-21.9394	-20.9215	-13.1364	-11.1038	-10.0663	-9.41683	-8.68838	-8.55724	-9.40624	-9.031
209.5	-21.1427	-23.3776	-20.7956	-20.394	-12.4527	-10.69	-9.40326	-8.80924	-7.98663	-8.57811	-9.33864	-9.06127
209	-20.2359	-22.5567	-19.9003	-20.3539	-12.0859	-10.2896	-8.69835	-8.28144	-7.45305	-8.16379	-8.92628	-9.20198
208.5	-19.0796	-22.0325	-19.1468	-19.8138	-11.7117	-9.60084	-8.00316	-7.89325	-6.89106	-7.59492	-9.07338	-9.37925
208	-18.3035	-20.4518	-18.1557	-19.3469	-11.1792	-8.82008	-7.22351	-7.87414	-7.14322	-7.31411	-9.30754	-9.21865
207.5	-16.491	-18.9227	-16.5771	-18.3782	-10.362	-8.83895	-7.39915	-7.7331	-6.041	-7.38505	-9.44982	-8.49921
207	-15.2028	-17.3678	-14.735	-16.39	-9.26718	-8.57043	-6.18379	-7.64312	-5.58367	-7.07467	-8.95065	-8.05455
206.5	-14.3066	-15.8588	-12.5427	-15.3527	-7.61932	-7.27254	-6.15107	-7.33673	-5.8415	-6.51274	-9.20396	-7.49682
206	-14.2434	-14.3749	-10.7157	-14.1633	-6.20225	-6.26008	-4.99874	-7.25473	-6.47028	-6.57352	-8.77946	-7.1182
205.5	-13.5094	-12.1374	-9.13286	-13.8712	-4.5105	-7.83937	-4.679	-8.46845	-5.29152	-5.91916	-8.57578	-6.64607
205	-11.5299	-10.9569	-9.03726	-12.5516	-4.87157	-7.45767	-4.51989	-9.04868	-4.00468	-5.59602	-8.16844	-6.59747
204.5	-10.7939	-10.1472	-8.06371	-11.0376	-2.71683	-6.14327	-4.46147	-9.80652	-2.45747	-4.99458	-8.83369	-7.0257
204	-9.23098	-7.34654	-7.44181	-10.7457	-2.61393	-7.1167	-3.3949	-10.9083	-1.84227	-4.11523	-7.71009	-6.55665
203.5	-7.06635	-4.2	-7.80874	-9.74498	-0.54347	-6.33987	-4.63415	-12.1296	-0.90955	-3.09766	-6.7763	-6.58553
203	-6.3704	-2.24948	-8.41728	-8.74712	-0.26601	-6.9327	-6.94162	-12.2506	-2.68738	-1.37158	-6.53472	-7.30879
202.5	-5.57477	-1.92077	-8.29582	-8.30888	0.951835	-6.26751	-6.53316	-11.5229	0.08836	-0.74756	-7.19932	-7.75014
202	-4.67349	-1.49422	-7.47812	-8.49978	2.45938	-6.63697	-5.24657	-9.62413	-0.62612	0.938644	-7.72339	-8.25583
201.5	-5.31865	-1.01054	-7.80705	-8.45659	1.67178	-5.36002	-5.86813	-8.4837	-1.15415	2.17331	-9.8102	-8.73243
201	-5.72205	0.930969	-8.24822	-7.73677	0.720106	-4.11078	-5.02489	-10.1423	-2.17364	3.92668	-9.58579	-8.95687
200.5	-6.92929	1.11415	-8.04786	-7.03875	0.166941	-2.42591	-6.50243	-9.4192	-3.18253	5.02622	-9.90423	-8.75445
200	-7.05233	-1.07914	-8.38614	-8.91362	-0.49476	-0.84985	-5.9257	-8.5347	-3.76925	4.28158	-9.9023	-10.6172
199.5	-7.04644	-0.20826	-9.07134	-9.34736	-0.59854	-0.82947	-5.93495	-7.46107	-3.05938	4.92619	-11.2	-11.3313
199	-6.28571	1.0375	-9.05891	-11.2855	-1.20555	-1.85701	-6.49862	-7.61652	-4.46786	5.54523	-10.8684	-11.7312
198.5	-5.61985	1.81577	-8.91733	-12.4089	-1.90937	-5.49229	-5.16918	-9.66674	-5.04213	4.21488	-9.58249	-10.6836
198	-4.80274	1.89054	-7.56677	-13.8542	-3.44292	-4.48876	-4.41109	-10.0561	-5.66784	4.87058	-9.24259	-9.98143
197.5	-5.53034	2.43185	-8.57165	-13.0184	-2.14835	-2.68192	-3.4862	-12.6725	-6.13584	4.55315	-9.6477	-9.38846
197	-6.61375	2.61394	-7.20636	-12.3708	-2.09264	-2.76859	-3.70355	-11.8873	-6.63082	4.82615	-10.5539	-7.91018
196.5	-7.0887	0.007231	-6.75666	-13.2795	-5.92012	-2.18834	-2.58898	-11.8318	-4.47032	2.12463	-11.9566	-8.38951
196	-7.51392	0.723987	-5.6777	-14.2561	-5.86242	-1.59944	-3.4058	-11.8287	-4.16888	-0.3585	-13.1588	-8.74811
195.5	-7.25076	2.87174	-4.40729	-15.5636	-6.64336	0.013714	-3.77276	-13.1704	-3.88084	-2.47256	-13.0186	-7.95718
195	-7.12116	4.28792	-3.40182	-4.56285	-6.58027	-0.47055	-4.16268	-13.9205	-3.05554	-2.42858	-10.3359	-8.6875
