# Supplementary material for: Stepwise assembly of α-hemolysin from intermediates to the mature pore in native erythrocytes
Source: J Cell Biol. 2026 Jan 12;225(3):e202506129. doi: 10.1083/jcb.202506129 (PMC12794805; doi:10.1083/jcb.202506129)
Supplement: Data S7 — shows values corresponding to the bar graph related to Fig. 5 G. [file jcb_202506129_datas7.pdf]

Heptamer	Octamer	Arc
63.66	31.13	5.19
