# Supplementary material for: Stepwise assembly of α-hemolysin from intermediates to the mature pore in native erythrocytes
Source: J Cell Biol. 2026 Jan 12;225(3):e202506129. doi: 10.1083/jcb.202506129 (PMC12794805; doi:10.1083/jcb.202506129)
Supplement: Data S8 — shows values corresponding to the bar graph related to Fig. 5 H. [file jcb_202506129_datas8.pdf]

| Pre-pore Ia | Pre-pore Ib | Pre-pore II | Pre-pore III | Pre-pore IV |
| --- | --- | --- | --- | --- |
| 26.95 | 13.19 | 19.23 | 13.06 | 27.53 |
