# Supplementary material for: Stepwise assembly of α-hemolysin from intermediates to the mature pore in native erythrocytes
Source: J Cell Biol. 2026 Jan 12;225(3):e202506129. doi: 10.1083/jcb.202506129 (PMC12794805; doi:10.1083/jcb.202506129)
Supplement: Data S9 — shows values corresponding to the bar graph related to Fig. 5 I. [file jcb_202506129_datas9.pdf]

Arc_4mer	Arc_5mer	Arc_6mer
5.47	89.63	4.89
