# Supplementary material for: Stepwise assembly of α-hemolysin from intermediates to the mature pore in native erythrocytes
Source: J Cell Biol. 2026 Jan 12;225(3):e202506129. doi: 10.1083/jcb.202506129 (PMC12794805; doi:10.1083/jcb.202506129)

Lane1 control RBC
Lane2 RBC pellet after toxin incubation
Lane3 supernatant from the toxin incubated sample
Lane4 protein marker
Lane5 Supernatant sample after heating

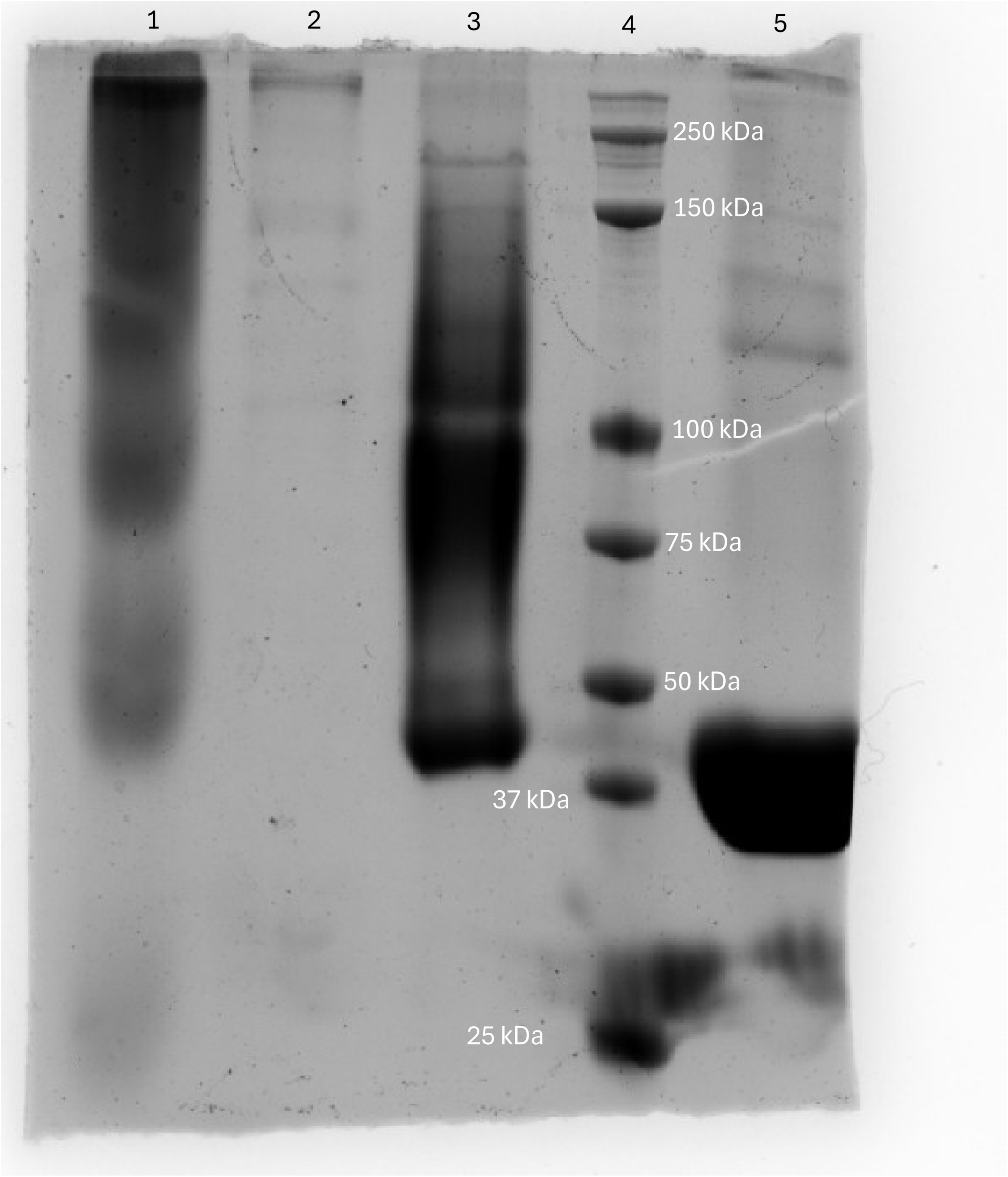

Supplement: SourceData F1 — is the source file for Fig. 1. [file jcb_202506129_sourcedataf1.pdf]
