# Supplementary material for: Stepwise assembly of α-hemolysin from intermediates to the mature pore in native erythrocytes
Source: J Cell Biol. 2026 Jan 12;225(3):e202506129. doi: 10.1083/jcb.202506129 (PMC12794805; doi:10.1083/jcb.202506129)

Lane 1	α-HL monomer	
Lane 2	X	
Lane 3	α-HL monomer	Used for figure
Lane 4	X	
Lane 5	α-HL monomer	
Lane 6	Marker	
Lane 7	α-HL monomer	
Lane 8	X	

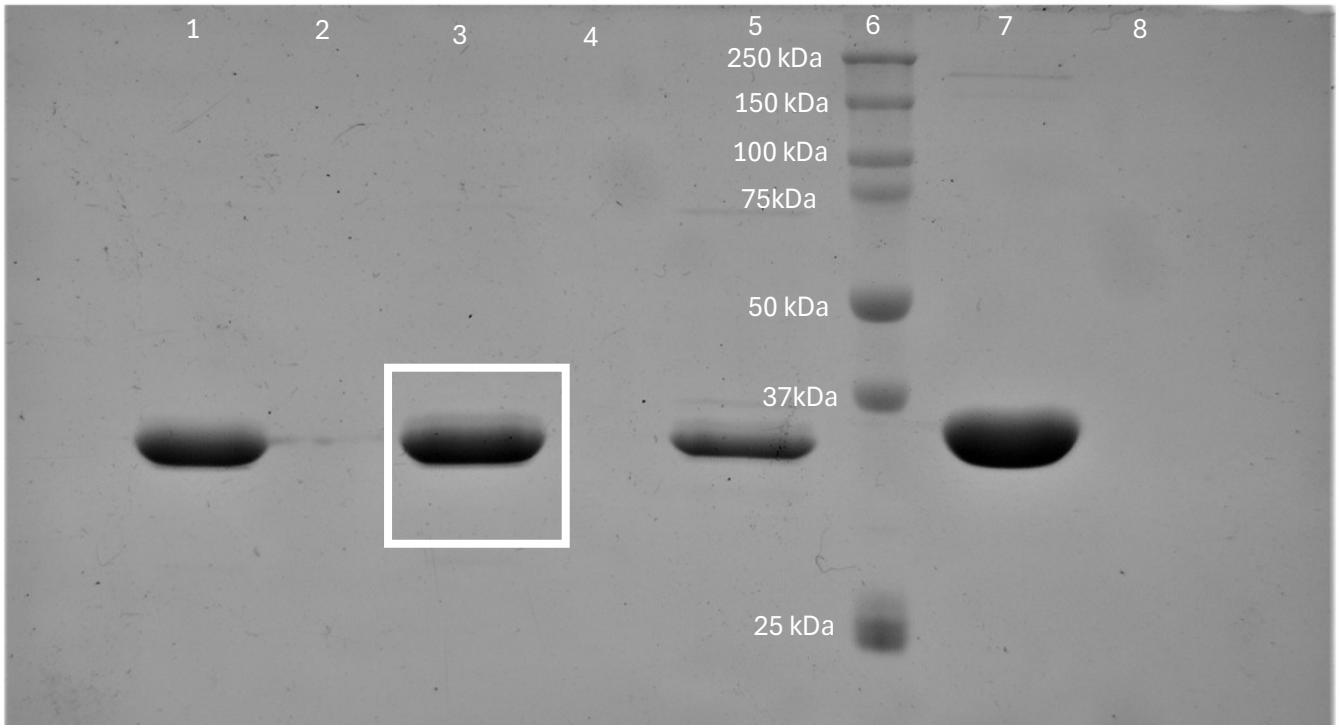

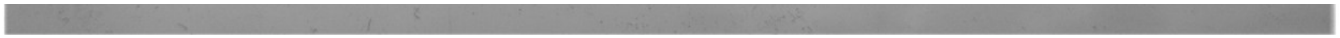

Supplement: SourceData FS1 — is the source file for Fig. S1. [file jcb_202506129_sourcedatafs1.pdf]
