# Supplementary material for: Stepwise assembly of α-hemolysin from intermediates to the mature pore in native erythrocytes
Source: J Cell Biol. 2026 Jan 12;225(3):e202506129. doi: 10.1083/jcb.202506129 (PMC12794805; doi:10.1083/jcb.202506129)

RBC pellet-lane 1
untreated RBC supernatant-lane 2
toxin-treated RBC pellet-lane 3
toxin-treated RBC supernatant-lane 4
protein marker-lane 5

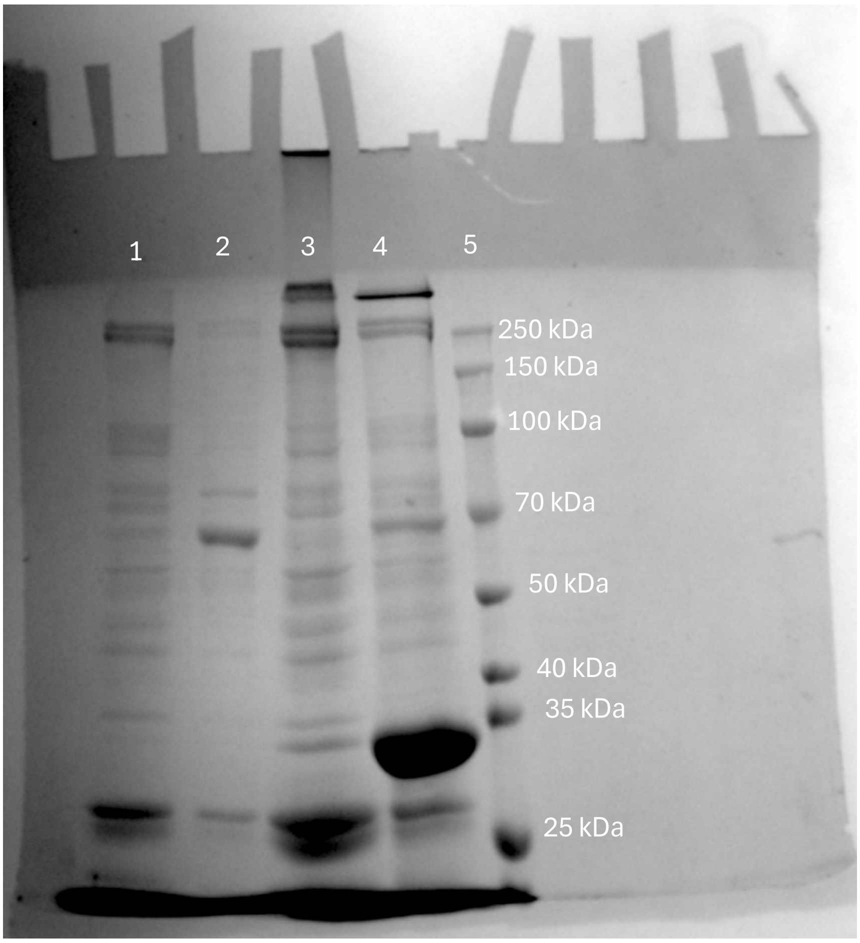

Supplement: SourceData FS4 — is the source file for Fig. S4. [file jcb_202506129_sourcedatafs4.pdf]
